# Artificial intelligence: A novel tool for diagnosing and managing kidney problems in pregnant women

**DOI:** 10.18502/ijrm.v23i5.19267

**Published:** 2025-07-29

**Authors:** Ahmad Shajari, Masoud Rostami, Vida Sadat Anoosheh Candidate

**Affiliations:** ^1^Department of Medical Sciences, Ali-Ebne-Abitaleb School of Medicine, Islamic Azad University, Yazd, Iran.; ^2^Department of Languages and Literature, Yazd University, Yazd, Iran.; ^3^Department of Ergonomics, School of Health, Student Research Committee, Shiraz University of Medical Sciences, Shiraz, Iran.

##  Dear Editor,

The health of pregnant women is a fundamental aspect of healthcare and medical services. During pregnancy, which involves significant physiological and hormonal changes, this issue becomes even more critical (1). Pregnancy not only causes considerable physical changes for the mother but also places substantial stress on various organs, particularly the kidneys. This additional burden can disrupt normal kidney function and lead to severe complications that threaten both maternal and fetal health (2). Renal disease during pregnancy is a serious medical concern as it may result in life-threatening complications such as renal failure, pregnancy-induced hypertension, preeclampsia (hypertension accompanied by other symptoms during pregnancy), premature labor, and intrauterine growth restriction (3).

Kidney diseases related to pregnancy often present with mild and nonspecific early symptoms that can easily be overlooked, making early diagnosis challenging. Symptoms like limb swelling, elevated blood pressure, or changes in urinary function may initially be subtle but can progress to severe complications if not diagnosed and managed promptly, posing serious risks to both mother and fetus (4). Moreover, traditional diagnostic methods -such as blood and urine tests, clinical examinations, and medical imaging- may have limited ability to detect these conditions accurately and in a timely manner. These methods often lack the capacity to predict disease progression precisely or to identify renal complications early enough, making timely diagnosis during pregnancy difficult (5).

Given the critical importance of early diagnosis and prediction of kidney disease progression in pregnancy, innovative and evidence-based solutions are urgently required. In this context, modern technologies such as artificial intelligence (AI)—specifically machine learning models and artificial neural networks—have shown promise as effective and innovative tools for diagnosing and managing kidney problems in pregnant women (6).

One of AI's greatest advantages is its ability to analyze and simulate complex datasets with high accuracy and provide earlier predictions. Machine learning algorithms can process diverse data points—including blood pressure, urinary protein levels, blood glucose, blood and urine test results, genetic information, and maternal medical history—to identify patterns that indicate an increased risk of kidney problems (7). This predictive capability enables healthcare providers to identify at-risk pregnant women and implement preventive strategies to halt the progression of kidney disease.

AI systems can also assist clinicians in medical decision-making by offering timely and effective treatment recommendations. Accurate identification of high-risk women is a major benefit of AI, allowing for earlier and more successful preventive and therapeutic interventions (8).

To effectively apply AI for diagnosing and managing kidney problems in pregnant women, relevant medical and clinical data such as blood pressure, blood and urine test results, history of kidney disease, diabetes, urinary tract infections, and lifestyle factors must first be collected. These data are then cleansed, normalized, and divided into training and testing datasets. Subsequently, machine learning algorithms such as decision trees, random forests, and artificial neural networks are trained to detect hidden and complex patterns to predict kidney disease risk. Finally, the models are evaluated using metrics like accuracy, sensitivity, and specificity to ensure their effectiveness in real clinical settings (9).

Following model development, testing and validation are essential. AI models must be assessed for accuracy, sensitivity, and specificity to determine whether they can reliably predict kidney problems. Validation uses real-world clinical and hospital data to confirm that the models perform well in practical situations (10).

The adoption of AI in the diagnosis and treatment of kidney disease during pregnancy has the potential to revolutionize healthcare. This technology aids clinicians in early identification of kidney disease, enabling timely and appropriate interventions that prevent serious complications such as renal failure, preterm labor, and fetal growth restriction. Beyond improving clinical outcomes, AI application in prenatal care can reduce treatment costs and enhance the overall quality of care.

Therefore, the integration of AI in this field represents a significant opportunity to optimize maternal health and reduce kidney-related complications during pregnancy, warranting serious consideration.
